# Vertical Nystagmus Recognition Based on Deep Learning

**DOI:** 10.3390/s23031592

**Published:** 2023-02-01

**Authors:** Haibo Li, Zhifan Yang

**Affiliations:** College of Electronic and Electrical Engineering, Shanghai University of Engineering Science, 333 Longteng Road, Shanghai 201620, China

**Keywords:** vertical nystagmus, deep learning, depthwise separable convolution, convolutional attention

## Abstract

Vertical nystagmus is a common neuro-ophthalmic sign in vestibular medicine. Vertical nystagmus not only reflects the functional state of vertical semicircular canal but also reflects the effect of otoliths. Medical experts can take nystagmus symptoms as the key factor to determine the cause of dizziness. Traditional observation (visual observation conducted by medical experts) may be biased subjectively. Visual examination also requires medical experts to have enough experience to make an accurate diagnosis. With the development of science and technology, the detection system for nystagmus can be realized by using artificial intelligence technology. In this paper, a vertical nystagmus recognition method is proposed based on deep learning. This method is mainly composed of a dilated convolution layer module, a depthwise separable convolution module, a convolution attention module, a Bilstm−GRU module, etc. The average recognition accuracy of the proposed method is 91%. Using the same training dataset and test set, the recognition accuracy of this method for vertical nystagmus was 2% higher than other methods.

## 1. Introduction

Benign paroxysmal positional vertigo (BPPV) is the most common vestibular peripheral vertigo, which is a transient induced vertigo when the head position changes to a specific position. The pathogenesis of BPPV has been widely recognized around the theory of canalithiasis and cupulolithiasis. At present, all kinds of induction tests and corresponding manual reduction therapy have been taken as the primary diagnosis and treatment method of BPPV in various hospitals, and these methods have achieved obvious effects [1,2]. For example, from August 2012 to August 2014, 175 patients with BPPV were diagnosed in the vestibular function examination room of the ENT Head and Neck Surgery Department of Xiangya Hospital of Central South University [3]. These patients, comprising 53 males and 122 females, were successfully treated by manual reduction. All patients were questioned about their medical history in detail, including vertigo attack, past history, and family history, and routine otological examination was performed. All patients were examined with the American VisualEyes infrared nystagmograph. The patients wore goggles to complete all body position tests in a dark room. In 175 patients with BPPV, vertical nystagmus was recorded in all patients.

Vertical nystagmus is a common neuro-ophthalmic sign in the field of vestibular medicine. Vertical nystagmus not only reflects the functional state of the vertical semicircular canal but also reflects the role of otoliths. According to the literature [4,5,6,7], medical experts can take nystagmus symptoms as the key factor to determine the cause of dizziness. At present, the clinical diagnosis of BPPV mainly depends on the specific displacement test to induce nystagmus in addition to the preliminary judgment of the involved semicircular canal according to the patient’s history characteristics. Then, according to different types of otolith reduction, the patients can obtain better therapeutic effect. Therefore, accurate nystagmus detection and analysis are the premise of correct diagnosis for BPPV and the key to ensuring efficacy.

Traditional observation is visual observation conducted by medical experts, which may be biased subjectively. Visual examination also requires medical experts to have enough experience to make an accurate diagnosis. In addition, people with dizziness may feel uncomfortable when trying to keep their eyes open completely, so their eyes may only remain partially open. Therefore, it is necessary to emphasize the observation of nystagmus to support clinical decision so as to enhance the diagnostic accuracy of medical experts [8]. Meanwhile, a practical method is needed to accurately detect nystagmus and provide results to medical experts.

Electronystagmography (ENG) is an image of the electric field changes around the eyeball when the eyeball is moving. The eyeball is a bipolar sphere. The cornea shows a positive potential relative to the retina and the retina shows a negative potential relative to the cornea. The two constitute an axis of potential difference. When the eyeball is in the emmetropic position, the potential difference between the cornea and the retina is about 1mV, and an electric field is formed on the head and face. This electric field changes its spatial phase when the eyeball moves. Placing an electrode on the skin of both sides of the eyeball, a voltage value can be drawn between the two electrodes. The voltage obtained was amplified with the principle of bioelectrical amplification, and recorded as an image. This is called electronystagmography, which reflects the change of the eye position. Visual observation of nystagmus is greatly limited, and it is difficult to analyze quantitatively. Accordingly, Henriksson [9] designed a special electronic electronystagmography machine and applied it to clinical practice. At present, electronystagmography is one of the important means for the localization diagnosis of the nervous system.

ENG has been applied to otology, mainly for the diagnosis of lesions around the vestibular system. At present, electronystagmography has been widely used in various clinical departments. Recording devices and technology have been greatly improved, especially the application of computers. The analysis of electronystagmography parameters has developed from naked-eye and manual analysis to automatic sampling quantitative analysis, which has promoted the research of electronystagmography and improved its application value.

Another method to measure eye movement is video measurement [10,11,12]. This method uses cameras to capture eye movement videos and uses relevant software to track pupil movements. With the development of computer vision technology, video ophthalmology has become a frequently used method [13]. Syahbana [14] proposed a method to obtain nystagmus waveform by visual measurement. This method estimates the eye movement by tracking the position of the patient’s eye pupil. In order to accurately estimate the position of the patient’s pupil, it is necessary to model the shape of the pupil. Generally, the existing research adopts the circular shape to approximate the pupil shape [15], such as Hough transform method [16]. However, the actual pupil shape is not a perfect circle. The approximate ellipse shape leads to a decline in the accuracy of pupil estimation. To solve this problem, Syahbana [14] proposed a pupil detection and tracking method based on the Mexican hat elliptical pattern, which can improve the accuracy of pupil position estimation.

It is very difficult to detect the vertical nystagmus with electronystagmograph (ENG). Most quantitative observations of human and animal optokinetic nystagmus (OKN) are conducted on the horizontal plane. It is generally agreed that using ENG to record the vertical movement of the eyeball leads to blinking artifacts. Iijima [17] thinks that high-speed videography (VOG) can replace the traditional ENG. If the detection device can be miniaturized and the recording time can be extended, the system can be widely used in high-speed eye movement image detection. VOG was widely used in the diagnosis of vertigo. However, the clinical manifestations of vertigo change with time. In this condition, VOG can be used in emergency and telemedicine diagnoses [18,19]. In such a different clinical environment, the challenges faced by VOG interpretation are not insignificant. Most emergency doctors have not received VOG equipment training, let alone the patients experiencing dizziness. Partly because of these problems, telemedicine solutions have emerged, allowing neuroscientists to quickly interpret VOG data remotely [20]. However, the number of neuro-otologists is not adequate and the implementation of telemedicine solutions is unrealistic. In this case, VOG analysis with automatic nystagmus detection is becoming a potential key solution for the future.

Charoenpong [21] proposed a method to detect involuntary eye movements with eye movement velocity. This method includes three main steps: pupil extraction, eye movement velocity calculation, and nystagmus detection. The accuracy of non-autonomous eye movement detection was 87.21%. The error is due to the inaccurate extraction of the pupil center. In practice, it is difficult to evaluate patients with videonystagmography (VNG) when their pupils are covered by drooping eyelids or eyelashes, and the interference of infrared light makes the situation worse [22]. Therefore, it is urgent to establish a nystagmus detection model.

With the development of technology, the detection system of nystagmus can be realized by using artificial intelligence (AI) technology. AI is an interdisciplinary approach, committed to data-driven experiential learning [23], which is considered as a potential solution to some medical diagnosis challenges. Zhang et al. [24] proposed a kind of nystagmus detection model based on optical flow technology, which can avoid interference caused by eyelash occlusion and pupil deformation. However, this model only provides a basic framework for the detection of nystagmus and cannot be directly applied to disease diagnosis. Lim et al. [25] developed a diagnosis decision support system for BPPV diagnosis using a two-dimensional convolutional neural network (2D-CNN) model. The results show that the system can detect nystagmus with a large number of training data, but this prediction ability is limited in the case of insufficient otological expert annotation data. Lu et al. [26] developed a new method for pupil location and iris distortion detection. This model has been verified in BPPV patients and has high sensitivity and accuracy in nystagmus detection and disease diagnosis. The first step of this method is to find the location of the pupil in each frame. The pupil location algorithm was used to locate the pupil center. 

The previous research tried to use deep learning model to predict the pupil position [27,28]. With the continuous improvement of deep learning, pupil detection has mainly used the data-driven mode. Tonsen et al. [29] designed a deep learning model based on an open-source dataset which contains 66 high-quality and high-speed videos [30] and then used the pre-trained model to mark the original video. 

On the basis of previous research, this paper designs a new method based on deep learning to detect vertical nystagmus so as to further improve the detection accuracy of vertical nystagmus. The innovation of this paper is to propose a new vertical nystagmus recognition method based on deep learning. We designed a new method of vertical nystagmus feature extraction and temporal feature recognition. The dilated convolution was used to obtain larger receptive field and more abstract features of vertical nystagmus. In order to reduce computational complexity, an improved depthwise-separable convolution structure was proposed to reduce the number of parameters which were needed for the calculation of vertical nystagmus feature extraction. L2 regularization strategy was added to the depthwise-separable convolution structure to solve the problem of over-fitting. Meanwhile, convolution attention mechanism was added to each depthwise-separable convolution operation to better obtain the channel features and plane space features of vertical nystagmus images. In order to improve the recognition accuracy, an improved GRU recognition model was proposed to capture the vertical nystagmus information at the critical moment. This paper is divided into five parts: The first part introduces the background of this research. The second part introduces the basic principle of the vertical nystagmus detection method. The third part introduces the experimental process and results. The fourth part is a comparison between this method and other methods. The last part is the conclusion.

## 2. Methods

**Figure 1:**
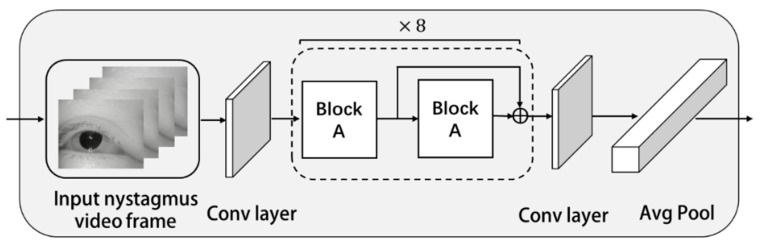
Network structure of feature extraction.

For the nystagmus video, it was converted into video frames and transmitted to feature extraction network for feature extraction. The network structure of feature extraction is shown in Figure 1.

It can be seen from Figure 1 that the video frame was first input to the convolution layer, and the output of the convolution layer was input to eight module groups which were repeated in series. Each module in the repeated module groups was combined by Block A and Block A with residual structure. There is a problem in the deep neural network. With the deepening of network layers and the increase in parameters, the network performance should be better. However, with the increasing layers, the network would degenerate quickly, and the training accuracy would decline. After adding the residual structure, the input was given more than one choice, which can solve the degradation problem. If the neural network learns that the parameters of one layer are redundant, it can choose to directly follow the “shortcut connection” curve and skip this redundant layer. After adding the residual module, the convergence speed of the model was accelerated, and the network can increase the layers. At the same time, the accuracy rate was also greatly improved. 

**Figure 2:**
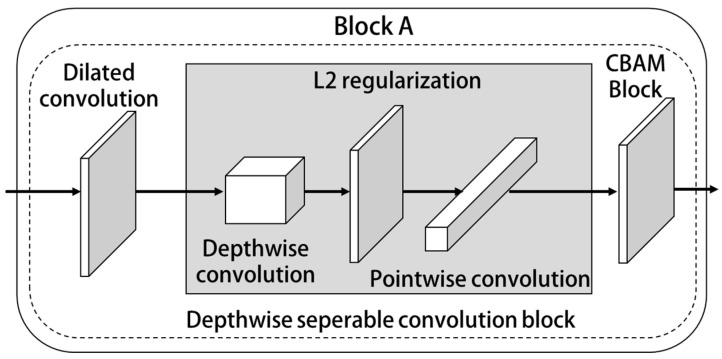
Block A structure.

In Figure 1, the structure of Block A is shown in Figure 2.

As can be seen from Figure 2, Block A is mainly composed of a dilated convolution layer module, depthwise separable convolution module and convolution attention module. These modules are introduced below.

### 2.1. Dilated Convolution Module

When using CNN for image processing, it is usually necessary to increase the receptive field of the model through multiple convolution and pooling operations. Pooling can reduce the size of the image, and using a convolution kernel can increase the receptive field; the stacking of multiple convolution kernels can also increase the receptive field. The feature map after convolution and pooling is relatively small, so the reduced feature map should be converted back to the original image size through the upsampling method. The main problem in this process is information loss. The pooling operation is irreversible. The information is lost when the image size is restored by upsampling the feature map. In order to avoid using pooling and other operations to expand the receptive field, we use the dilated convolution module [31] in Block A instead of the traditional max-pooling and structured convolution, which can increase the receptive field and keep the size of the feature map consistent with the original image.

Generally, when $F:Z^{2}\to R$ is a discrete function, $\Omega_{r}={\left[-r,r\right]}^{2}⋂Z^{2}$, $k:\Omega_{r}\to R$ is the convolution kernel with the size of ${\left(2r+1\right)}^{2}$. Then discrete convolution * is defined as Equation (1).

(1) $$\left(F\;*\;k\right)\left(p\right)=\sum F\left(s\right)k\left(t\right)$$

Then, taking *l* as expansion factor, convolution ${*}_{l}$ is defined as Equation (2).

(2) $$\left(F{*}_{l}k\right)\left(p\right)=\sum_{s+lt=p}F\left(s\right)k\left(t\right)$$

where ${*}_{l}$ is dilated convolution with a dilated factor of *l*. In addition, dilation convolution can be used systematically to obtain multi-scale context information. It can increase the size of each receptive field without increasing the number of model parameters. Therefore, we can get more receptive fields and abstract features by using dilated convolution.

### 2.2. Depthwise Separable Convolution Module

The principle of depthwise separable convolution is to divide a standard convolution layer into two parts, namely, depthwise convolution and point convolution. Each input channel of the depthwise convolution has an independent convolution kernel, which is equivalent to collecting the characteristics of each channel. The number of input channels and output channels are consistent, so there is no increase or decrease in the dimension of depthwise convolution. Point convolution realizes the function by using 1×1 convolution. It collects the characteristics of each point and can be used to increase or reduce dimensions.

To solve the problem of over fitting, L2 regulation was added to the depthwise separable convolution. L2 regulation is a classical method which was used to solve the over fitting problem in neural networks [32]. It can constrain and adjust the coefficients to 0. L2 regulation added $\Omega \left(\omega \right)$ to the original objective function to limit and constrain the fitting ability of the model. In Equations (3) and (4) [32], *X* is sample data, *y* is sample data label, *ω* is the weight coefficient.

(3) $$L\left(\omega ,X,y\right)=L_{1}\left(\omega ,X,y\right)+\lambda \Omega \left(\omega \right)$$

(4) $$\Omega \left(\omega \right)=\sum_{j}\omega_{j}^{2}$$

When the value of the objective function is the smallest, the weight coefficient can be obtained. It is shown in Equation (5).

(5) $$\underset{\omega }{\min}L_{1}\left(\omega ,X,y\right)$$

To control the complexity of the model, the constraint condition is shown in Equation (6).

(6) $$\sum_{j}\omega_{j}^{2}\leq C$$

**Figure 3:**
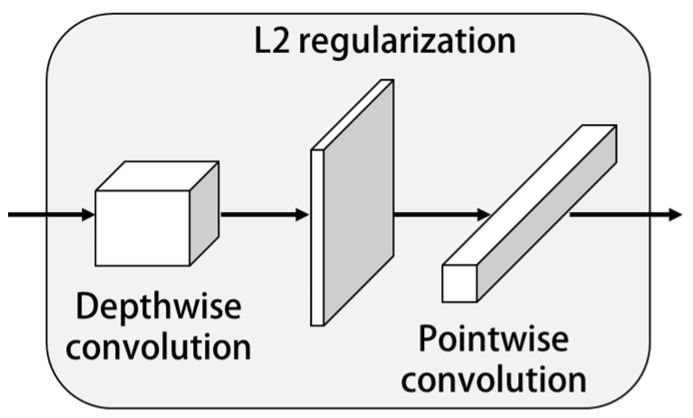
Regularization strategy to prevent over fitting.

That is, all the sum of the squares of ω should be less than or equal to *C*. Therefore, the goal of regularization is to minimize the value of $L_{1}$. Meanwhile, the constraint condition of Equation (6) should be met. As shown in Figure 3, the L2 regularization strategy was added to the depthwise separable convolution structure. 

### 2.3. Convolutional Attention Module

**Figure 4:**
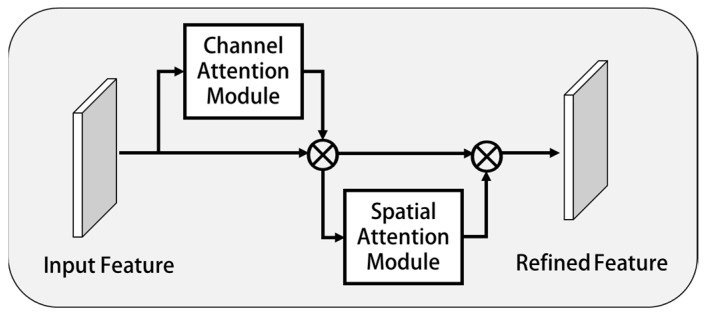
Convolution attention mechanism.

This paper introduces the convolutional block attention module (CBAM) [33] to improve performance. CBAM can infer attention feature map from any given feature map along the two non-interference aspects of space and channel. Then, the attention feature map was added to the input feature map so as to achieve the goal of fine-tuning the input feature map. CBAM uses two submodules, channel attention and spatial attention, to extract the features of space and channel. As shown in Figure 4, the feature map generated by any convolutional network at runtime was taken as the input feature graph $F\in R^{C\times H\times W}$, and then successively communicated with the channel attention module $M_{C}\in R^{C\times 1\times 1}$ and spatial attention module $M_{S}\in R^{1\times H\times W}$. It is shown in Equations (7) and (8) [33].

(7) $$F^{\prime }=M_{C}\left(F\right)⨂F$$

(8) $$F^{\prime \prime }=M_{S}\left(F^{\prime }\right)⨂F^{\prime }$$

where ⨂ represents the Hadamard product between elements, and $F^{\prime \prime }$ is the final output result.

The calculation process of these two submodules was analyzed below.

#### 2.3.1. Channel Attention Submodule

**Figure 5:**
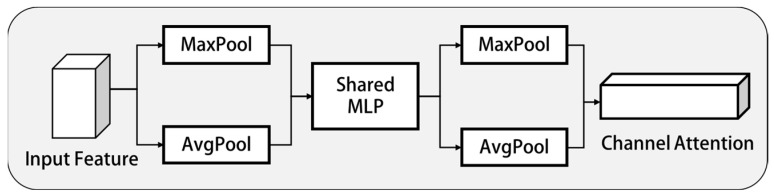
Channel attention module.

This module made use of the relationship between channels to build channel attention feature map. The goal of the channel attention module was to give more attention to areas with higher value in the picture. In order to make the channel attention feature map information more valuable, it is necessary to simplify and filter the spatial information of the input feature map. For the reduction and filtering of this part of information, average pooling (AvgPool) was usually used as the tools. MaxPool can also obtain important evidence about object features from different perspectives, so it can be used to obtain better channel attention feature map. AvgPool and MaxPool would affect the result. AvgPool reduces the variance of the estimated value caused by the limited neighborhood size; MaxPool can reduce the deviation of estimated mean value caused by the error of convolution parameters. Therefore, AvgPool and MaxPool are simultaneously used in channel attention to improve the feature extraction ability of the model, as shown in Figure 5.

In the way mentioned above, AvgPool and MaxPool were used to extract the main information in the spatial information at the same time so as to obtain the characteristic graph $F_{avg}^{c}$ of AvgPool and $F_{max}^{c}$ of MaxPool. Then two feature maps were input into a shared multi-layer perceptron network to obtain channel attention feature map $M_{C}\in R^{C\times 1\times 1}$. At the same time, the ratio *r* was adjusted to reduce the number of parameters, while the size of the active function of the hidden layer was set to $R^{C/r\times 1\times 1}$. Then, the above results were added and processed to obtain the output characteristic diagram, as shown in Equation (9).

(9) $$\begin{array}{c} M_{C}\left(F\right)=\sigma \left(MLP\left(AvgPool\left(F\right)\right)\right)+MLP\left(MaxPool\left(F\right)\right)\\ =\sigma \left(W_{1}\left(W_{0}\left(F_{avg}^{c}\right)\right)\right)+W_{1}\left(W_{0}F_{max}^{c}\right) \end{array}$$

Among them, *σ* represents the activation function, $W_{0}\in R^{C/r\times C}$, $W_{1}\in R^{C\times C/r}$.

#### 2.3.2. Spatial Attention Submodule

**Figure 6:**
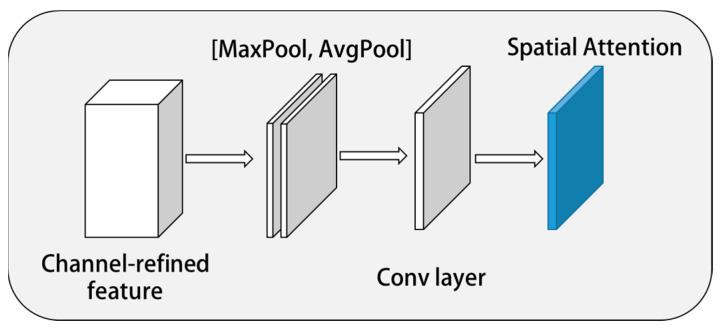
Spatial attention module.

This module is shown in Figure 6. It constructs the spatial attention feature map through the spatial relationship on the two-dimensional plane. Compared with channel attention, the spatial attention submodule pays more attention to where the features are effective, thus supplementing the channel features. In the spatial attention submodule, average pooling and maximum pooling were used for processing, and the results were linked to obtain efficient feature description. This method has been proved to be effective for marking key information areas. In the obtained relational feature description, the convolution layer was used to judge the spatial areas that need to be concerned and ignored, so as to obtain the spatial attention feature map $M_{s}\left(F\right)\in R^{H\times W}$. First, different pooling methods were used to obtain an average pooling feature map $F_{avg}^{s}$ and maximum pooling feature map $F_{max}^{s}$. Then the two were connected and convolved to obtain the two-dimensional spatial attention mechanism submodule. It is shown in Equation (10).

(10) $$\begin{array}{c} M_{s}\left(F\right)=\sigma \left(f^{7\times 7}\left(\left[AvgPool\left(F\right);MaxPool\left(F\right)\right]\right)\right)\\ =\sigma \left(f^{7\times 7}\left(\left[F_{avg}^{s};F_{max}^{s}\right]\right)\right) \end{array}$$

In the formula, *σ* is the activation function; $f^{7\times 7}$ represents the convolution of a 7×7 convolution kernel.

Overall, CBAM is a simple and effective attention module. There is not a large amount of convolutional structure inside the CBAM module, and there is a small amount of pooling layers and feature fusion operation. This structure avoids a large amount of computation caused by convolution multiplication, which makes the module have low complexity and small computation. Given an intermediate feature map, the attention weight was deduced along the space and channel dimensions in CBAM, and then multiplied with the original feature map to adaptively adjust the features. CBAM is a lightweight general module which can be seamlessly integrated into convolutional neural network (CNN) architecture and can conduct end-to-end training with the CNN. After CBAM processing, the new feature map obtains the attention weight of the channel and spatial dimensions, which greatly improves the connection of each feature in the channel and space, and is more conducive to extracting the effective features of vertical nystagmus. Adding CBAM to the nystagmus detection task can improve the representation ability of the model. This method can effectively reduce the interference of invalid targets and improve the recognition effect of nystagmus.

**Figure 7:**
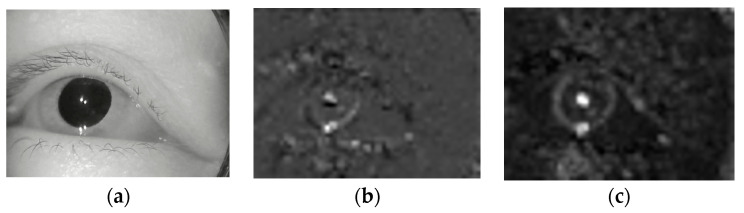
Comparison of feature maps between the general feature output and the attention mechanism output. (**a**) Original image. (**b**) The general feature output. (**c**) The attention mechanism output.

Using the basic CBAM attention submodule, we can adjust the weight of eye movement semantic features, apply greater weight to more important features, enhance the correlation between the model and nystagmus features, and reduce redundant features. Furthermore, we compared the feature maps between the attention mechanism output and the general feature output, as shown in Figure 7.

From Figure 7, through software analysis, the feature map of attention mechanism output has more activation values in the pupil area where nystagmus occurs.

### 2.4. Activation Function

**Figure 8:**
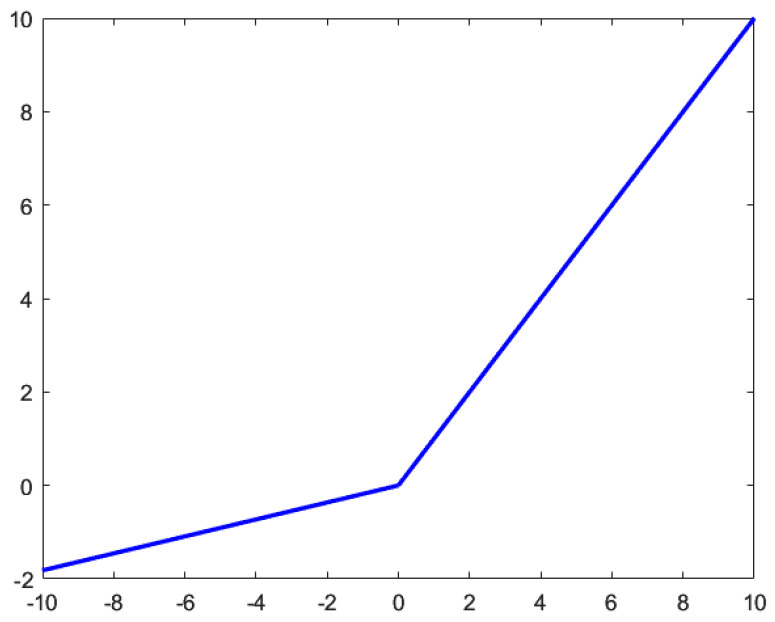
Leaky ReLU activation function ($a_{i}$ = 5.5).

The activation function is the key point of nonlinear learning ability in neural network structure. The ReLU activation function can effectively alleviate the gradient dispersion phenomenon and becomes the mainstream choice of most activation functions. However, with the increase in the number of training rounds of the network, the corresponding weights of some neurons cannot be updated, resulting in neuron death, and ReLU discards the negative value information in the feature map during feature extraction. The mean value of ReLU output value is always greater than zero, which is not conducive to the expression of network learning ability. The characteristics of the Leaky ReLU activation function can solve the problem of ReLU and effectively extract negative value feature information. The mathematical form is shown in Equation (11) [34].

(11) $$y_{i}=\left\{\begin{array}{c} \frac{x_{i}}{a_{i}},x_{i}<0\\ x_{i},x_{i}\geq 0 \end{array}\right.$$

where $x_{i}$ represents the output of layer *i*; $y_{i}$ represents the output of layer *i* after nonlinear transformation; $a_{i}$ is a fixed parameter in layer *i*, and its range is (1, +∞). The Leaky ReLU activation function is shown in Figure 8. As the negative axis of the Leaky ReLU activation function is a function with a small slope, it can initialize neurons, avoid neuron death, and increase the extraction of negative feature information. Negative value information comes from the feature extraction calculation process. During the experiment, when $a_{i}=5.5$ in the Leaky ReLU activation function, the classification effect of the Leaky ReLU activation function is better than that of the ReLU activation function.

### 2.5. BiLSTM−GRU Module

**Figure 9:**
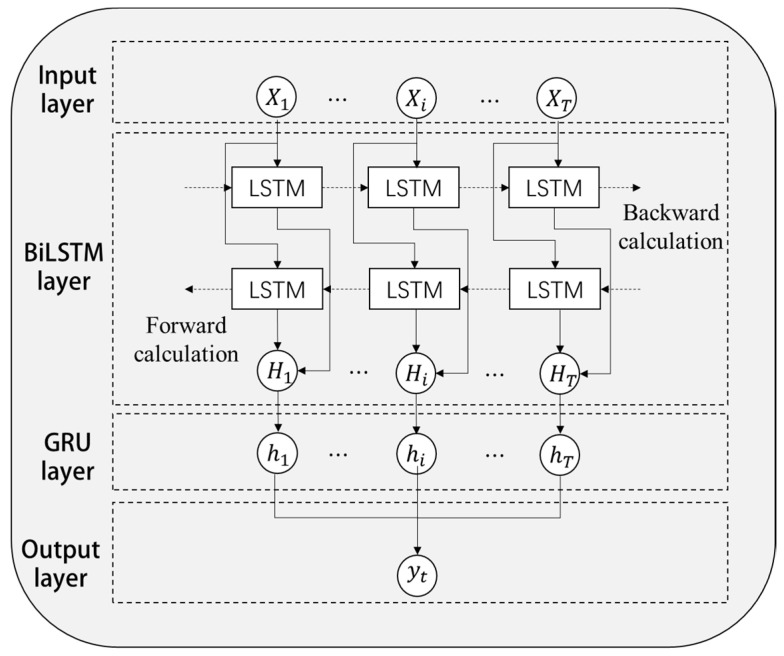
Structure of BiLSTM-GRU module.

When traditional recognition models deal with multivariate time series, it is often difficult to capture the complex changes of vertical nystagmus, resulting in low recognition accuracy. To solve this problem, this paper uses the prediction method of BiLSTM–GRU combined model to identify the characteristics of vertical nystagmus. Firstly, BiLSTM was used to extract time-series features in both directions, and then GRU neural network was used to further study the change rules of bi-directional time-series features to accurately capture vertical nystagmus information at critical moments. The BiLSTM−GRU module is mainly composed of an input layer, BiLSTM layer, GRU layer, and output layer. Its structure diagram is shown in Figure 9.

It can be seen from Figure 9 that the input layer takes the output of the feature extraction module as the input of the BiLSTM−GRU module. The input sequence at time *t* is shown in Equation (12).

(12) $$X_{t}={\left[X_{1},\cdots ,X_{i},\cdots ,X_{T}\right]}^{T}$$

where $X_{t}$ is the input sequence at time *t*, and *T* is the time steps.

The forward sequence ${\left[X_{1},\cdots ,X_{i},\cdots ,X_{T}\right]}^{T}$ was entered in the forward layer of the BiLSTM layer and the hidden state ${\vec{H}}_{i}$ of the *i*-th layer was obtained by calculation. The reverse sequence ${\left[X_{T},\cdots ,X_{i},\cdots ,X_{1}\right]}^{T}$ was entered in the back layer and the hidden state ${\overset{\leftarrow }{H}}_{i}$ of the *i*-th layer was also obtained by calculation. The *i*-th hidden state value of BiLSTM at time *t* was confirmed by calculation through Equation (13).

(13) $$H_{i}=\alpha {\vec{H}}_{i}+\beta {\overset{\leftarrow }{H}}_{i}$$

where α, β are constants and α+β=1.

Finally, the result in Equation (14) was taken as the bidirectional timing sequence extracted at time *t*.

(14) $$H_{t}={\left[H_{1},\cdots ,H_{i},\cdots ,H_{T}\right]}^{T}$$

Bidirectional sequence $H_{t}$ output from BiLSTM was used as the input sequence of GRU layer for further learning.

(15) $$h_{i}=GRU\left(H_{i-1},H_{i}\right)$$

(16) $$h_{t}={\left[h_{1},\cdots ,h_{i},\cdots ,h_{T}\right]}^{T}$$

where $h_{i}$ is the hidden layer state of the *i*-th GRU layer at time *t*, and $h_{t}$ in Equation (16) is the hidden layer state of the GRU layer at time *t*.

Output layer: the Leaky ReLU function was adopted as the activation function to output the final predicted value at the final time *t*, which is recorded as:

(17) $$y_{t}=\sigma \left(h_{t}\right)$$

where σ is the activation function.

## 3. Experimental Verification of the Designed Method

**Figure 10:**
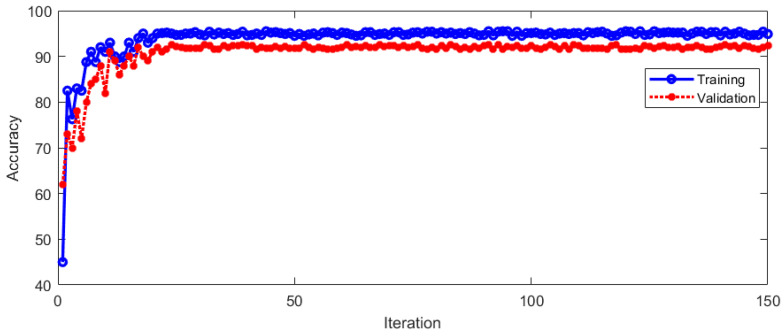
Training and verification of the model.

The dataset used in this paper is from the Eye & ENT Hospital of Fudan University. The vertical nystagmus training data and test data were annotated by ophthalmologists of Affiliated Hospital of Fudan University in Shanghai of China. The equipment used for nystagmus video capture was the eye movement recorder of Shanghai Zhiting Medical Technology Co., Ltd. The vertical nystagmus video is 640 × 480 pixels and 60 fps. The collected data came from 1090 patients, and 21,743 segments of vertical nystagmus video were collected. The collected data were labeled by the doctors of the hospital to form a test and validation dataset; 80% of the samples in the dataset were used for model training, and 20% were used for test verification. The training results of the proposed model and the verification results are shown in Figure 10.

From Figure 10, it can be seen that the proposed model has a good effect in the training and verification process. With the increase in the number of training iterations, the classification accuracy of the model continues to improve. The model tends to be stable after 24 iterations. The recognition accuracy of vertical nystagmus when the model was stable during training and verification is shown in Table 1.

**Figure 11:**
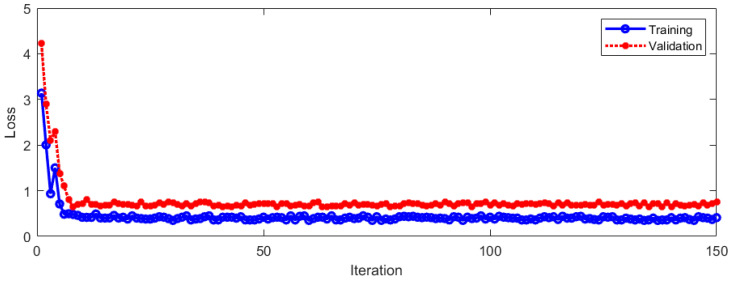
LOSS during training and verification.

The LOSS of the model during training and verification is shown in Figure 11.

**Figure 12:**
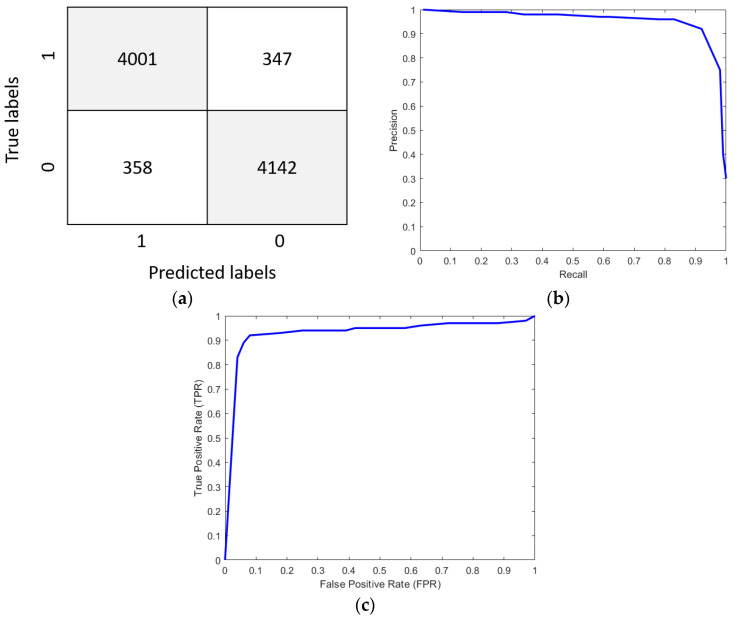
Model evaluation. (**a**) Confusion matrix. (**b**) PR curve. (**c**) ROC curve.

It can be seen from Figure 11 that the LOSS of the model gradually drops to a stable state during the training and verification process with the increase in training iterations. When the LOSS was stable, it was in a lower numerical range. In order to further evaluate the algorithm, Figure 12 shows the fusion matrix, PR curve, and ROC curve.

In Figure 12a, 0 indicates no nystagmus and 1 indicates nystagmus. It can be seen from Figure 11 that the proposed method can identify vertical nystagmus more accurately.

In order to inspect the effect of each module of the proposed algorithm on the overall performance of the model, an ablation experiment was carried out. The experimental results are shown in Table 2.

As can be seen from Table 2, the introduction of convolution attention module significantly improved the classification accuracy. This shows that the introduction of attention mechanism in the network can better extract nystagmus motion characteristics and spatiotemporal information. Other modules also improve the classification accuracy.

## 4. Comparison of Feature Extraction Method Replacement

**Figure 13:**
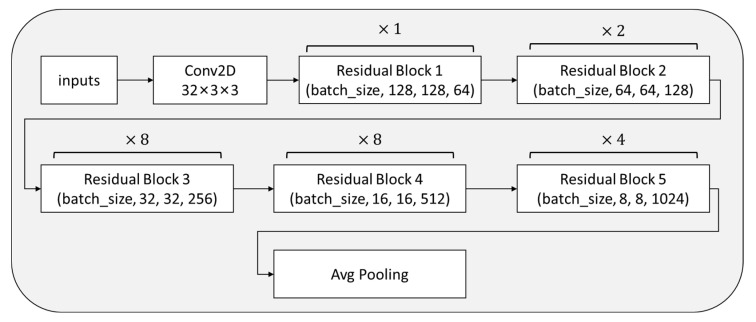
Structure of feature extraction module.

In the process of model design, we designed another feature extraction method to compare the recognition effect of vertical nystagmus. The feature extraction network module structure is shown in Figure 13.

**Figure 14:**
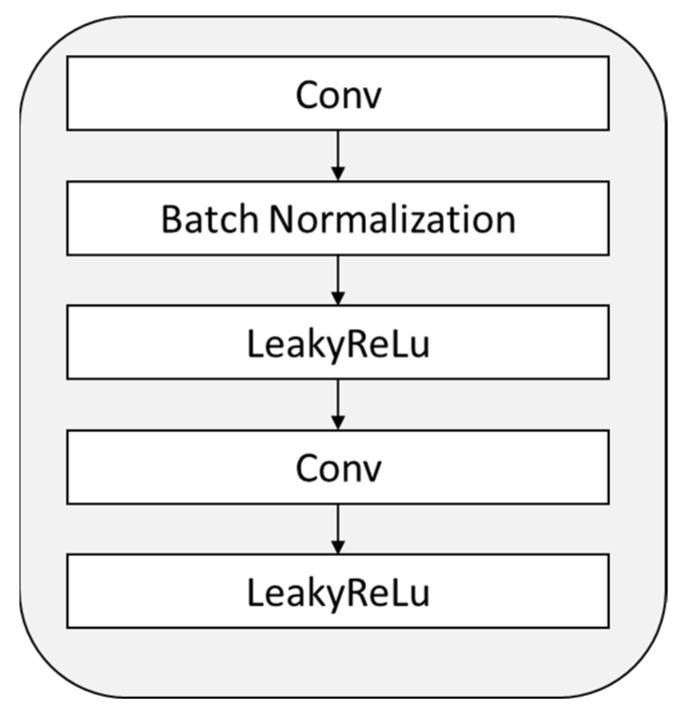
Structural diagram of residual block.

The feature extraction model mainly includes convolution layer, residual block, and average pooling layer. The structure of each residual block is shown in Figure 14.

**Figure 15:**
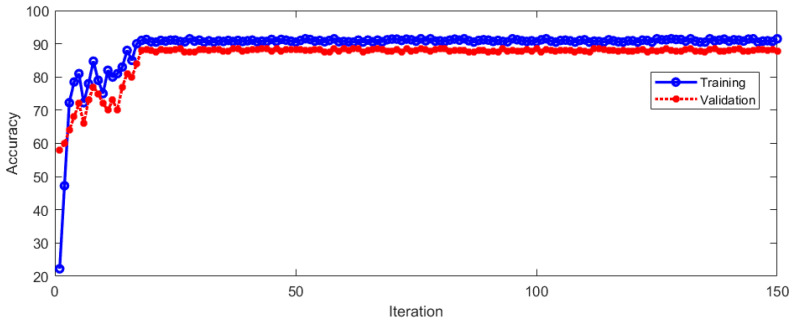
Recognition accuracy in training and verification process.

This vertical nystagmus recognition method was named Method 2 with this feature extraction method. For Method 2, we used the same training dataset for training and the same verification set for test. The training and verification process is shown in Figure 15.

**Figure 16:**
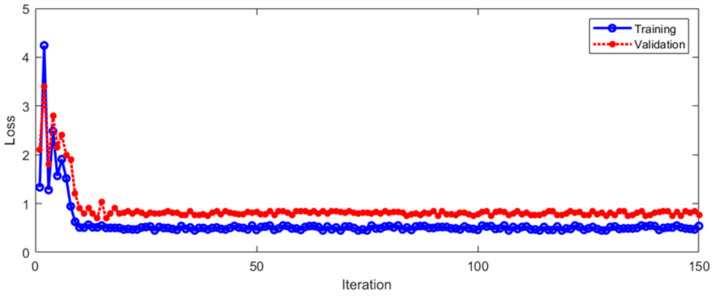
The LOSS of Method 2 during training and verification.

It can be seen from Figure 15 that the recognition accuracy is constantly improved during the training and verification process. With the increase in iterations, the recognition accuracy tends to be stable. The model started to be stable after 21 iterations. This shows that this method is feasible. The LOSS during training and verification is shown in Figure 16.

**Figure 17:**
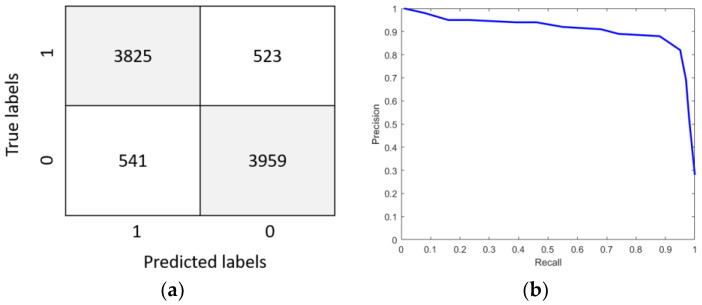

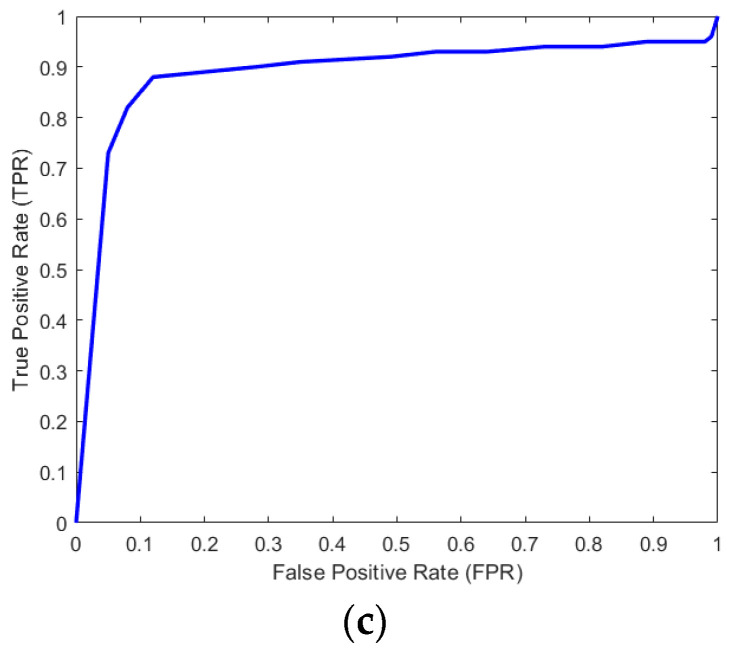
Method 2 evaluation. (**a**) Confusion matrix. (**b**) PR curve. (**c**) ROC curve.

It can be seen from Figure 16 that the LOSS of Method 2 gradually drops to a stable state in the process of training and verification. With the increase in iterations, the curve remains in a small numerical range. In order to further evaluate Method 2, Figure 17 shows the fusion matrix, PR curve, and ROC curve.

**Figure 18:**
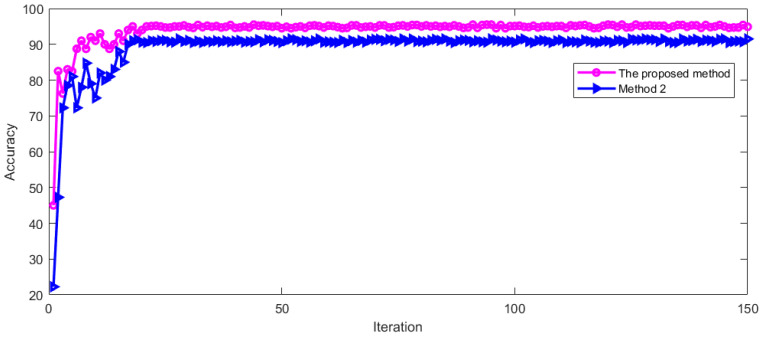
The accuracy comparison of two methods in training process.

In Figure 17a, 0 indicates no nystagmus and 1 indicates nystagmus. It can be seen from Figure 16 that method 2 can also identify vertical nystagmus efficiently. Then the recognition accuracy of Method 2 was compared with the proposed method. The comparison result is shown in Figure 18.

It can be seen from Figure 18 that the recognition accuracy of vertical nystagmus is constantly improving. With the increase in training iterations, the recognition accuracy tends to be stable. After 24 iterations, the process started to be stable. The average recognition accuracy of the two methods is shown in Table 3 after the model recognition tends to be stable.

**Figure 19:**
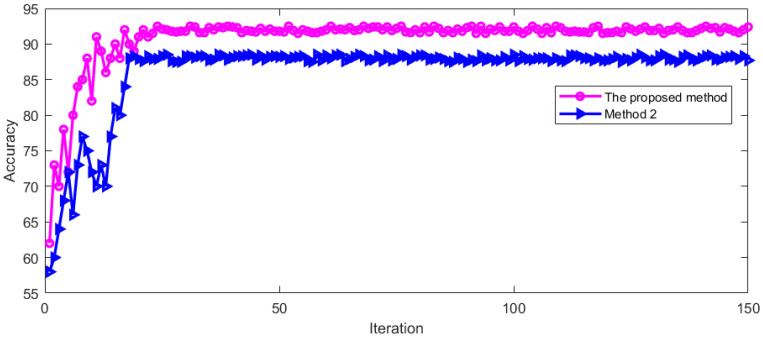
The recognition accuracy of two methods in the test set.

It can be seen from Table 3 that the proposed method has a high recognition accuracy of vertical nystagmus. The vertical nystagmus recognition accuracy of the two methods in the test set is shown in Figure 19.

As can be seen from Figure 19, the recognition accuracy of vertical nystagmus continues to improve and become stable with the increase in iterations. The process started to be stable after 24 iterations. When the recognition accuracy tends to be stable, the average recognition accuracy of the two methods is shown in Table 4.

It can be seen from Table 4 that the proposed method has a high recognition accuracy on the test set after the model is stable.

## 5. Comparison with Other Methods

**Figure 20:**
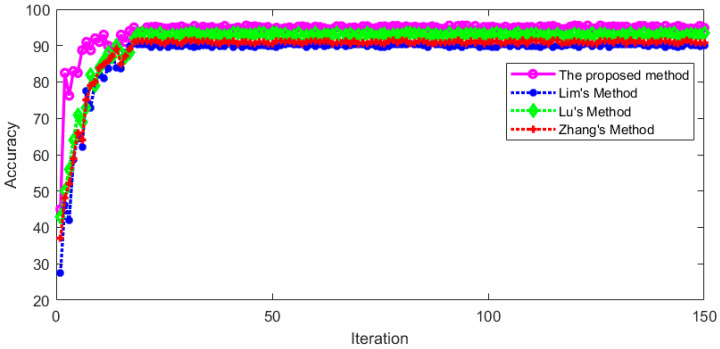
The recognition accuracy for four methods in training set.

**Figure 21:**
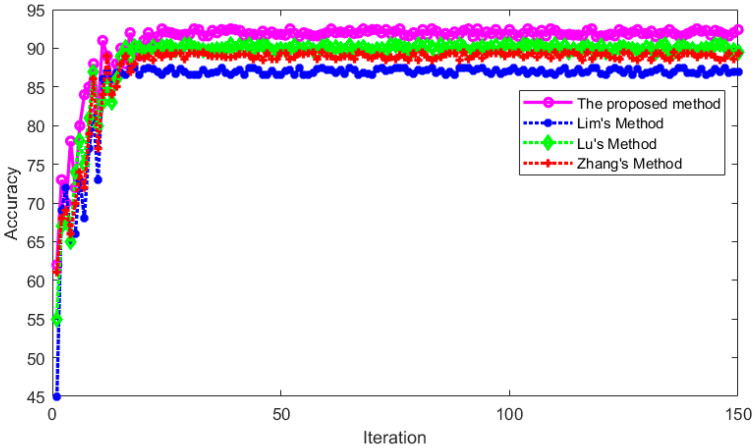
The recognition accuracy for four methods in test set.

The proposed method was compared with Lim’s method [25], Lu’s method [26], and Zhang’s method [24]. These methods used the same training set for training and used the same verification set for testing. The recognition accuracy during training and testing is shown in Figure 20 and Figure 21, respectively.

It can be seen from Figure 20 and Figure 21 that the recognition accuracy of these methods tends to be stable with the increase in iterations during the training and testing process, which indicates that these methods are feasible for vertical nystagmus recognition. After the model recognition is stable, the average recognition accuracy in the training set and verification set is shown in Table 5 and Table 6, respectively.

**Figure 22:**
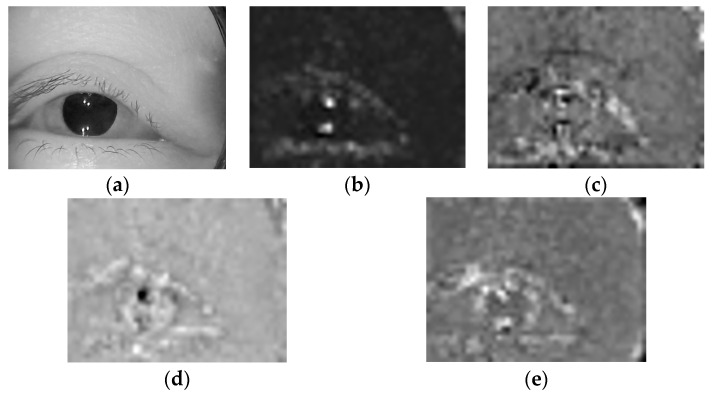
Original image and intermediate results. (**a**) Original image. (**b**) Proposed method. (**c**) Lim’s method. (**d**) Lu’s method. (**e**) Zhang’s method.

It can be seen from Table 5 and Table 6 that the proposed method has a relatively high recognition accuracy for vertical nystagmus, which indicates that the proposed method has a good effect on vertical nystagmus recognition. Further, we extracted sample images from original videos and the intermediate results in the main processes, as shown in Figure 22.

From the results of program statistics, the intermediate feature map of the proposed method has the most activation values. 

Compared with other methods, the proposed method does not need to locate the pupil. Zhang’s method needs to calibrate the pupil and combine it with Hough transform and trajectory tracking based on template matching. Lu’s method also needs to mark the position of the pupil center and use the pre-training model to label the original video. Lim’s method used an algorithm based on the center of gravity to track the pupil center. Circular Hough transform was used to detect elliptical pupil. If the pupil was found, an edge detection and ellipse fitting algorithm would be used to locate the center of the pupil. Compared with other methods, the proposed method simplifies the processing process. In the data processing, Lu’s method used data enhancement and Zhang’s method compressed video data. The proposed method and Lim’s method directly used the original video clip. This reduced the calculation steps. From the experimental results, the proposed method can further improve the accuracy of vertical nystagmus recognition. In the future, the recognition accuracy may be further improved, which requires the efforts of more researchers.

## 6. Conclusions

In this paper, a vertical nystagmus recognition method was proposed based on deep learning. This method is mainly composed of dilated convolution layer module, depthwise separable convolution module, convolution attention module, Bilstm−GRU module, etc. Dilation convolution was used systematically to obtain multi-scale context information. It can increase the size of each receptive field without increasing the number of model parameters, avoiding down sampling and reducing the information of feature map. To solve the problem of over fitting, L2 regulation was added to the depthwise separable convolution. In addition, CBAM was introduced to improve the performance. CBAM can infer attention feature maps from any given feature map along the two non-interference aspects of space and channel. This paper used the Leaky ReLU activation function. As the negative half-axis of the Leaky ReLU activation function is a function with a small slope, it can initialize neurons, avoid neuron death, and increase the feature extraction of negative value information. Finally, the BiLSTM−GRU module was used to classify and recognize the extracted features. From the experimental results, the proposed method can effectively identify vertical nystagmus. Compared with other methods, the proposed method has a higher recognition accuracy.

## Figures and Tables

**Table 1:** Recognition accuracy after model stabilization.

Recognition Accuracy	Stage
94.96%	Training
92.03%	Validation

**Table 2:** Influence of different modules in the model.

Condition	Accuracy
Model (no dilated convolution layer module)	84.89%
Model (no depthwise separable convolution module)	85.92%
Model (no convolution attention module)	83.35%
Model (no Bilstm module)	89.57%
Model (full version)	92.03%

**Table 3:** The average recognition accuracy of two methods.

Recognition Accuracy	Method
94.96%	The proposed method
91.03%	Method 2

**Table 4:** The average recognition accuracy of two methods in the test set.

Recognition Accuracy	Method
92.03%	The proposed method
87.97%	Method 2

**Table 5:** The average recognition accuracy of these methods in training set.

Method	Data Processing Methods	Feature Processing Methods	Morphological Method	Recognition Accuracy
Proposed method	Original video clip	Spatiotemporal sequence	Not required	94.96%
Lim’s method	Original video clip	Grid images	Required	89.99%
Lu’s method	Data augmentation	Phase correlation	Not required	92.95%
Zhang’s method	Video condensation	Optical flow guide	Required	90.98%

**Table 6:** The average recognition accuracy of these methods in test set.

Recognition Accuracy	Method
92.03%	Proposed method
87.04%	Lim’s method
90.03%	Lu’s method
89.01%	Zhang’s method

## Data Availability

The data that support the findings of this study are available from the corresponding author upon reasonable request.
